# Rethinking the treatment of chronic fatigue syndrome—a reanalysis and evaluation of findings from a recent major trial of graded exercise and CBT

**DOI:** 10.1186/s40359-018-0218-3

**Published:** 2018-03-22

**Authors:** Carolyn E. Wilshire, Tom Kindlon, Robert Courtney, Alem Matthees, David Tuller, Keith Geraghty, Bruce Levin

**Affiliations:** 1 School of Psychology, Victoria University of Wellington, New Zealand, P.O. Box 600, Wellington, New Zealand; 2 Irish ME/CFS Association, Dublin, Ireland; 3 London, UK; 4 Perth, Australia; 5 School of Public Health, University of California, Berkeley, California USA; 6 School of Health Sciences, University of Manchester, Manchester, UK; 7 Department of Biostatistics, Columbia University, New York, USA

**Keywords:** Chronic fatigue syndrome, Myalgic encephalomyelitis, Graded exercise therapy, Cognitive behavioral therapy

## Abstract

**Background:**

The PACE trial was a well-powered randomised trial designed to examine the efficacy of graded exercise therapy (GET) and cognitive behavioural therapy (CBT) for chronic fatigue syndrome. Reports concluded that both treatments were moderately effective, each leading to recovery in over a fifth of patients. However, the reported analyses did not consistently follow the procedures set out in the published protocol, and it is unclear whether the conclusions are fully justified by the evidence.

**Methods:**

Here, we present results based on the original protocol-specified procedures. Data from a recent Freedom of Information request enabled us to closely approximate these procedures. We also evaluate the conclusions from the trial as a whole.

**Results:**

On the original protocol-specified primary outcome measure - overall improvement rates - there was a significant effect of treatment group. However, the groups receiving CBT or GET did not significantly outperform the Control group after correcting for the number of comparisons specified in the trial protocol. Also, rates of recovery were consistently low and not significantly different across treatment groups. Finally, on secondary measures, significant effects were almost entirely confined to self-report measures. These effects did not endure beyond two years.

**Conclusions:**

These findings raise serious concerns about the robustness of the claims made about the efficacy of CBT and GET. The modest treatment effects obtained on self-report measures in the PACE trial do not exceed what could be reasonably accounted for by participant reporting biases.

## Background

For some time now, the officially recommended treatments for chronic fatigue syndrome (CFS) in many countries have been graded exercise therapy (GET) and cognitive behavioural therapy (CBT). In an effort to provide high quality evidence of the efficacy of these treatments, White and colleagues undertook a large randomised trial, informally referred to as the PACE trial [1]. Reports from the PACE trial concluded that GET and CBT were moderately effective treatments for CFS, both leading to recovery in over a fifth of patients [2–7]. The trial’s size and its promotion as a success have made it enormously influential in the attempt to treat CFS [8].

However, there are some significant concerns with the published reports of the trial. First, the outcomes and analyses presented in these reports did not always follow the procedures set out in the original published protocol [1]. Since the purpose of a trial protocol is to prevent ad hoc modifications that may unduly favour the study hypotheses, it is important to carefully scrutinise the justification for these changes and how they may have influenced outcomes. Also, it is unclear whether some of the trial’s conclusions about treatment efficacy were fully justified by the evidence. Here, we present several new analyses of the trial data, using methods that align with those specified in the original trial protocol, and drawing on data recently made available as part of a Freedom of information application ([9]). This dataset, henceforth referred to as the FOIA dataset, is available to the public (see Declarations section for instructions on how to download the dataset). We also explore several other aspects of the findings not considered in the published reports, and evaluate the conclusions from the trial as a whole.

### Summary of the PACE trial

PACE was a large randomised trial whose primary aim was to assess the effectiveness of GET and CBT as treatments for CFS (early publications refer to it as a “randomised controlled trial”, but “randomised trial” is more appropriate, given that several nuisance variables were not fully controlled across trial arms, e.g., contact hours). Participants were 641 adults with mild-to-moderate CFS defined by the Oxford criteria [10]: the principal symptom must be fatigue, which must have had a definite onset, resulted in significant disability, and have persisted for at least six months. Participants also had to score 65 or less on the Short-Form Health Survey Physical Function subscale [11]. Also, they had to report experiencing at least six of the 11 fatigue items on the Chalder Fatigue Questionnaire (CFQ [12]), as “more than” or “much more than” than prior to illness.

Participants were randomised into four groups. All were offered at least three medical consultations. The first group, which we will call *Control*, received no further treatment (the trial publications use the term *Specialised Medical Care*). The other groups received up to 15 therapy sessions over 36 weeks. One group received CBT, one GET, and the fourth group received a novel treatment, Adaptive Pacing Therapy. Both the CBT and the GET interventions were built upon a behavioural/deconditioning model of CFS. This model proposes that there is no major ongoing disease process underlying CFS - only deconditioning due to recent inactivity, and its various consequences. When patients attempt to increase their activity, they experience normal fatigue, stiffness and other symptoms, which they misinterpret as signs of continuing disease. The patients then become more focused on their symptoms, and fearful of further activity, creating a self-perpetuating cycle [2]. The GET programme was designed to help CFS patients overcome this purported fear of exercise and intense symptom-focusing through graded exposure to exercise, and thereby also reverse any deconditioning that had occurred. Participants were asked to choose an aerobic activity they enjoyed, and to gradually increase the duration and intensity of that activity under the supervision of a therapist. The CBT programme had similar aims, but addressed the fear of activity, maladaptive illness beliefs and symptom focusing using a combination of CBT and practical activities ([2], p. 825). Participants were encouraged to view their symptoms as arising from anxiety, intense symptom focusing and/or deconditioning. The sessions addressed fears about exercise and other “unhelpful cognitions” that may perpetuate symptoms, and encouraged participants to try gradually increasing their activity ([2], p. 825).

Adaptive Pacing Therapy, in which patients were advised not to exceed a certain level of activity, was created specifically for the trial. Results for this trial arm did not differ significantly from those for the Control arm for any of the outcomes considered in this article. Consequently, we will not discuss them further here.

### Primary outcomes

The primary outcome for the trial, as specified in the trial protocol published in 2007, was the percentage of patients who fulfilled the specified criteria for overall improvement 52 weeks after randomisation [1]. Two measures contributed to the definition of improvement: self-rated fatigue, measured using the Chalder Fatigue Questionnaire [12], and self-rated disability, measured using the SF-36 Physical Function subscale [11]. The minimum levels of improvement required on each of these two measures are given in Table 1 (Definition A). However, in May 2010, several months after data collection was complete, this primary outcome measure was replaced with two continuous measures: fatigue and physical function ratings on the two scales described above (see [13, 14] for details). According to the researchers, the changes were made “before any examination of outcome data was started...” ([13], p. 25).

**Table 1:** Definitions of improvement and recovery specified in the trial protocol [1], and those used in the final trial reports [2, 4]. Improvement was the primary outcome measure specified in the protocol. Recovery was a secondary measure

	Definition A: Specified in trial protocol	Definition B: Used in published reports
Overall Improvement	Minimum score of 75 on the 100-point SF-36 physical function scale *or* a score increase of 50% or more.	At least an 8 point increase in the 100-point SF-36 physical function scale.
	Of the 11 fatigue items on the Chalder Fatigue Questionnaire (CFQ), three or fewer rated as worse/much worse than prior to illness OR the total items rated worse/much worse dropped by at least a 50%.	At least a 2 point decrease on the 33-point CFQ (Likert scoring method).
Recovery	Minimum score of 85 on the 100-point SF-36 physical function scale.	Minimum score of 60 on the 100-point SF-36 physical function scale.
	Of the 11 items on the CFQ, three or fewer rated as worse/much worse than prior to illness.	Maximum score of 18 on the 33-point CFQ.
	Overall health self-rated as “very much better” on the Clinical Global Impression scale [50].	Overall health self-rated as “much better” or “very much better” on the Clinical Global Impression scale.
	The final “caseness” criterion was met if the patient no longer fulfilled: The Oxford case definition of CFS; the CDC criteria [51]; AND the London ME criteria [52]. (As determined by a non-blinded assessor).	The revised “caseness” criterion was met if ANY of the following applied: a) the patient did not meet the standard Oxford case definition; OR b) on the CFQ, they rated less than six of the 11 fatigue items as being worse than prior to illness; OR c) their SF-36 Physical Function score was greater than 65.

*CFQ* Chalder Fatigue Questionnaire

In 2011, the first major publication from the trial reported results based on this new primary outcome [2]. It was found that, following treatment, scores on both these continuous measures improved in all groups, but significantly more so in the CBT and GET groups than in the other groups. In the 2011 publication, rates of overall improvement were also reported; however, these were not based on the protocol-specified definition, but rather on a very different, and much more generous, one: Definition B in Table 1. By this new definition, 59% of CBT participants and 61% of GET participants were classed as having improved overall [2]. However, 45% of Control participants did so too. Results for the original protocol-specified definition of improvement - Definition A in Table 1 – do not appear in any peer reviewed publications from the trial (which number in the double digits [15]).

### Rates of recovery

An important secondary outcome specified in the trial protocol was the proportion of patients who met the specified definition of recovery at the end of the trial [1]. The definition of recovery presented there considered each participant’s scores on two key self-rated measures (fatigue, physical function), one further measure of overall self-rated improvement and finally, whether the participant still met various CFS case definitions. The complete definition of recovery is given in Table 1 (Definition A). However, results for this outcome never appeared in published reports. Instead, a 2013 paper reported recovery rates based on a much more generous definition of recovery (Definition B in Table 1) [4]. According to these new criteria, 22% of patients in each of the CBT and GET groups qualified as recovered, but only 7% in the Control group. The difference in recovery rates between the CBT/GET groups and the Control group was statistically significant. The PACE investigators have not specified when the decision to change the definition of recovery was made, except to say it was “before the analysis occurred” [16]; the change does not appear in any documentation prior to the final publication, and there is no published evidence that it was approved by the trial steering committee.

### Other outcome measures

A number of other secondary outcome measures were collected at 52 weeks, including several additional subjective outcomes, and also four objectively scored measures, which are described further below. During the course of the trial, data for a range of adverse events and outcomes were also collected; these are also described briefly below.

The four objectively scored measures examined at 52 weeks were: 1) distance walked in six minutes; 2) fitness (VO_2_max, estimated using the step-test method); 3) days lost from work during the six-month period following the primary endpoint; and 4) the percentage of participants receiving illness/disability benefit during that same period. In the 2011 primary trial report, only one of these outcomes was reported: walking speed [2]. Here, 69% of the GET group completed the test, and walked approximately 10–12% farther in six minutes than the 74% of Controls who completed the test. This small difference was statistically significant (based on an available case analysis), but given the high and uneven drop-out rate for these outcome measures, this result should be treated with caution. The CBT group did not walk significantly farther than Controls. Results for the other objective outcomes were not reported until some years later, and then only in summary form [3, 6]. H.owever, none appear to be associated with significant treatment effects. For the fitness measure, a simple one-way analysis of variance performed on the summary data extracted from ([6], Figure 2) failed to reveal a significant effect of treatment group, F(3,425) = 0.368, *ns*. For the employment loss measure, a similar analysis of the summary data in ([3], Table 2) also failed to reveal a significant treatment effect, F(3, 636) = 0.23, *ns*. Finally, for illness/disability benefit data, a binary logistic regression performed using the summary data in ([3], Table 3) did not reveal any significant treatment effect, χ^2^(3) = 0.00, ns.

**Table 2:** Outcomes at 52 weeks and long-term follow-up, excluding patients who completed any additional sessions of GET or CBT. Confidence intervals were only available for the follow-up phase

Measure	Group	N	52 weeks (mean scores for subgroup)	Long-term follow-up (means, 95%CIs)
SF-36 Physical Function Scale	Control	49	56.8	62.6 *(54.6, 70.6)*
	CBT	88	61.5	64.2 *(58.6, 69.8)*
	GET	95	62.8	62.5 *(57.1, 67.9)*
Chalder Fatigue Scale (“Likert” scoring method)	Control	49	22.6	18.7 *(16.2, 21.2)*
	CBT	88	20	17.9 *(16.1, 19.7)*
	GET	95	19.7	18.7 *(17.1, 20.3)*

*CIs* confidence intervals

The adverse events measures collected during the trial included: serious adverse events (death, hospitalisation, etc.); serious deterioration (a broader category that included a serious adverse event, sustained decrease in self-reported physical function or overall health, or withdrawal due to worsening); and non-serious adverse events. Serious adverse events were significantly more prevalent in the GET group (8%) than in the Control group (4%); there were no other statistically significant group differences.

### Long-term follow-up

A mail survey was conducted at least two years after randomisation (median 31 months [7]:). Survey response rates were 72%, 74% and 79% for the Control, CBT and GET groups respectively. Participants were again asked to complete the trial’s primary fatigue and physical rating scales, and several other questionnaires. A 2015 paper reported the results for the fatigue and physical function measures, again treating them as separate, continuous variables [7]. Analyses of these measures, based on an available case approach, failed to yield any significant effects of treatment group. However, the investigators did not view this negative result as a cause for concern at all. They argued that many patients in the Control and Adaptive therapy trial arms had received some CBT or GET after the conclusion of the main trial, and this could explain why they had since improved to the level of the other patients.

### Current analyses

The primary objective of our reanalyses was to examine how the trial outcomes would have looked if the investigators had adhered to their published protocol. Specifically, we were interested in analysing results for the primary outcome set out in that document: overall improvement rates. We also calculated recovery rates based on the definition outlined in the protocol. Results from this latter analysis have been published elsewhere [17, 18], but here we present more complete details of our method and findings. Finally, we explored the published data on long-term outcomes to examine whether they had been contaminated by patients’ post-trial therapy experiences, as the PACE researchers hypothesised.

## Methods

Using the FOIA dataset, we first calculated rates of improvement at the primary 52-week endpoint according to the definition specified in the trial protocol (Definition A in Table 1). We used an intent-to-treat approach, again as specified in the protocol: if the 52-week score was missing, that case was counted as a non-improver (there were no missing scores at baseline; missing scores had been replaced with scores at screening as described in [14]). However, for comparison, we also repeated the analysis based on an available case sample: participants with missing scores at 52 weeks were simply excluded from the dataset.

Based on the methods stipulated in the published protocol, we performed a logistic regression analysis on the binary outcome data from all four treatment arms. Where appropriate, we also performed pairwise comparisons between each of the two key treatment groups (CBT and GET), and the Control group, correcting for the total number of planned comparisons. The trial protocol lists six planned comparisons [1]. The statistical analysis plan, published some years later, lists only five [14]. Here, we report outcomes based on both scenarios. No method of correction was specified in the trial protocol, but in the statistical analysis plan, the Bonferroni method was stipulated [14], so this was the method we applied. All omnibus analyses (that is, all analyses examining the overall effect of treatment group on outcomes) included the adaptive pacing therapy group, because it forms part of the trial design. However, specific results for this group are not detailed here.

The protocol specified that various stratification variables would also be included in the primary outcome analysis (e.g., treatment centre, therapist, presence/absence of co-morbid depression). These variables were not available in the FOIA dataset, so we were unable to include them. Nonetheless, they were approximately evenly distributed across groups, and therefore their inclusion would be unlikely to change outcomes substantially [2]. Also, our team has previously shown that for one of the published logistic regression analyses (that for recovery rates based on Definition B in Table 1), replicating the analysis without the stratification variables had a negligible effect on the outcome of the analysis [18].

We also calculated recovery rates based on the definition specified in the trial protocol (Definition A in Table 1). Results from this analysis have been published elsewhere [17], but here we present more complete details of our method and findings. In the published protocol, it was not explicitly specified that an intent-to-treat approach would be applied, so we present results based on both an intent-to-treat approach (according to the definition above) and an available case approach (again, according to the definition above). Our definition of recovery closely approximated Definition A from the trial protocol, but may have been marginally more generous: in determining whether the final CFS “caseness” criterion was met, we considered only the Oxford case definition (the other case definitions were not available in the FOIA dataset). However, it is unlikely that this change impacted substantially on recovery rates, and if it had, its likely effect would have been to further reduce recovery rates for the CBT and GET groups relative to the other two groups (the maximum effect it could possibly have had was to exclude a further three individuals each from the CBT and GET “recovered” groups, and none from the Control group. This is the number of individuals that were excluded from the “recovered” group when these two alternative caseness criteria were added to the recovery definition used in [4]). We then performed a logistic regression analysis incorporating the binary recovery data from all four treatment arms. Where appropriate, we performed planned pairwise comparisons according to the procedures set out above for the primary outcome analysis.

Finally, to explore the PACE investigators’ hypothesis that long-term treatment effects may have been obscured by patients’ post-trial treatment choices, we isolated the long-term self-rated fatigue and physical function scores for those patients who did not receive any post-trial CBT or GET. The relevant individual patient data are not available in the FOIA dataset, so a systematic reanalysis could not be performed. However, since the relevant summary data are reported in [7], see Supplementary materials, Table C], we were able to perform a simple one-way analysis of variance examining the effect of original treatment allocation on long-term outcomes in this subgroup.

## Results

**Fig. 1:**
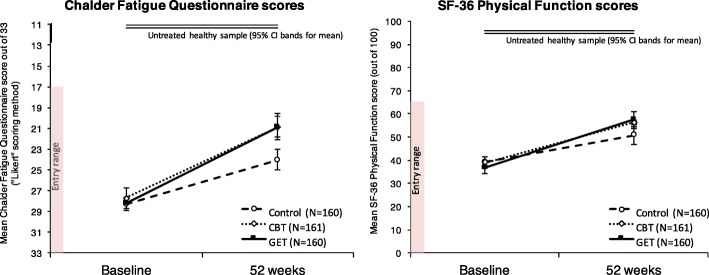
Intent-to-treat means for fatigue and physical function ratings, the two measures that contributed to the criterion for improvement specified in the published protocol (Definition A in Table 1). Estimates of healthy performance for the fatigue and physical function measures are based on previously published samples that further excluded the elderly (over 60), and those with a significant medical condition (95% CI bands = upper and lower bounds of 95% confidence interval). The relevant normative data for the Chalder Fatigue Questionnaire were obtained from [48], and those for the SF-36 physical function scale were obtained from [49]. In the case of the SF-36 scale, the healthy sample was highly negatively skewed, so medians are reported. The median score for this sample was 100 (95% confidence intervals: 100,100)

Figure 1 shows intent-to-treat means and confidence intervals for the two self-rated measures that contributed to the definition of improvement, alongside estimates of performance in healthy controls. A number of the Chalder Fatigue Questionnaire scores needed to calculate these rates of improvement were missing from the FOIA dataset; however, in every such case, the outcome could be inferred from other data available in the FOIA set. Based on the protocol-specified definition of improvement, 20% of CBT patients and 21% of GET patients improved, and 10% of the Control patients. These percentages accord with those calculated by the investigators and posted to the Primary Investigator’s institutional website shortly after the researchers were directed to release the data under FOI legislation ([19]; these results were never formally published and the statistical analyses specified in the original trial protocol were never performed).

There was a statistically significant effect of treatment on improvement rates, χ^2^(3) = 14.24, *p* = .003. The *p*-value associated with the contrast between CBT and Control was *p* = .015 and that for the contrast between GET and Control was *p* = .010. If we take into account all six planned comparisons listed in the protocol, the Bonferroni-adjusted *p* threshold for both pairwise comparisons is 0.008. Neither comparison reaches this threshold. The situation is not much improved if we consider only the five planned comparisons listed in the subsequent statistical analysis plan ([14]); the *p* threshold is 0.010. The comparison between GET and Control just reaches this threshold, but the comparison between CBT and Control does not.

The percentage of participants with missing outcomes at 52 weeks was small (5.2% across all trial arms). Nevertheless, to explore the impact of counting drop-outs as non-improvers, we repeated our calculations based on an available case sample. Using this definition, 11% of Control participants improved, compared to 22% and 21% of CBT and GET participants respectively. There was again a statistically significant overall effect of treatment group on improvement rates, χ^2^(3) = 15.02, *p* = .002. The *p*-value associated with the contrast between CBT and Control was *p* = .010 and that for the contrast between GET and Control was *p* = .011. However, once more, neither of these outcomes survives Bonferroni correction based on the number of planned comparisons specified in the trial protocol (corrected threshold *p* value = .008). Even using the looser criterion based on the statistical analysis plan (*p* = .010), the comparison between CBT and Control only just reaches the threshold of 0.01 and the comparison between GET and Control does not.

In addition to overall improvement rates, the trial protocol identifies rates of improvement on each of the two major contributing criteria – self-rated fatigue and physical function - as primary outcomes in their own right. So we analysed these outcomes in the same manner as above, using an intent-to-treat approach as specified in the protocol. Rates of protocol-specified improvement on the SF36 physical function criterion were 44% for the Control group, 48% for the CBT group, and 61% for the GET group. The overall effect of treatment arm was significant, χ^2^(3) = 16.31, *p* = .001. The *p*-value associated with the contrast between CBT and Control was *p* = .34 and that for the contrast between GET and Control was *p* = .002. The comparison between GET and Control survives correction for multiple comparisons (irrespective of whether one assumes five or six planned comparisons) but that between CBT and Control does not.

Rates of protocol-specified improvement on the CFQ criterion were 13% for the Control group, 26% for the CBT group, and 24% for the GET group. There was also a statistically significant effect of treatment on rates of improvement on the fatigue criterion, χ^2^(3) = 13.19, *p* = .004. The *p*-value associated with the contrast between CBT and Control was *p* = .004 and that for the contrast between GET and Control was *p* = .015. The former remains after correcting for multiple comparisons, but the latter does not.

### Recovery rates

Using the protocol-specified definition of recovery, and applying an intention-to-treat approach, the rates of recovery were 7%, 4% and 3% for the CBT, GET and Control groups respectively. Applying an available-case approach, these rates were 8%, 5%, and 3% respectively. In neither instance was there a statistically significant effect of treatment on recovery rates (*p* values were 0.14 and 0.10, respectively, for the intent-to-treat and available case approaches).

### Long-term outcomes

Out of those who responded to the long-term follow-up, 43% of the Control participants had received no further CBT or GET after the completion of the trial. This was also the case for 74% and 75% of the respondents from the CBT and GET arms respectively. Considered together, this subset of participants was perhaps slightly less severely affected than the remaining patients: they scored slightly better on the primary physical function and fatigue scales at 52 weeks than those who opted for further treatment (physical function: 61.3 vs. 48.1; fatigue: 23.9 vs. 25.9). However, at 52 weeks, the pattern of scores across treatment arms was the same as for the sample as a whole: the CBT and GET participants in our subset rated their fatigue as slightly lower and their physical function slightly higher at 52 weeks than the Control participants. In this respect, our subsample may be considered reasonably representative of the sample as a whole.

Table 2 provides arithmetic means for the two major self-report outcome measures for this subset of patients (i.e., those who received no further treatment). The pattern of results presented here mirrors that obtained for the entire cohort: the small group differences apparent on these measures at 52 weeks are no longer evident at long-term follow-up. A one-way analysis of variance revealed that there were no statistically reliable effects of treatment group on either outcome measure (Physical function: F(3,291) = 0.70, *ns*; Fatigue F(3,291) = 0.17, *ns*). If we repeat the analyses, adding in those cases who received some additional therapy sessions, but less than the minimum 10 considered by the investigators to be an “adequate” dose ([7], p.1071), the outcome does not change (Physical function: F(3,384) = 1.85, *ns*; Fatigue F(3,384) = 0.86, *ns*). Consequently, the disappearance of group differences at long-term follow-up cannot be attributed to the effects of additional post-trial therapy.

## Discussion

### Discussion of new results

Our reanalyses of the trial data based on the published protocol generated some troubling findings. First, scores on the protocol-specified primary outcome measure — improvement in self-reported fatigue and physical function – were numerically higher for the CBT and the GET groups than for the Control group. However, these differences did not pass the threshold for statistical significance after correcting for the number of planned comparisons specified in the trial protocol. Using a more lenient correction (assuming only five planned comparisons), outcomes are only marginally more positive: the comparison between GET and Control just reaches this threshold, but the comparison between CBT and Control does not. Of course, our analyses did not incorporate a number of important stratification variables that were unavailable in the FOIA dataset. However, it appears unlikely that their inclusion would substantially alter the result, and our analyses remain the closest approximation to the originally specified one that has ever been published. Our findings suggest that, had the investigators stuck to their original primary outcome measure, the outcomes would have appeared much less impressive.

Improvement rates for self-rated fatigue and physical function considered individually did yield some statistically significant findings, which suggests that the interventions were somewhat specific in the way they altered patients’ illness perceptions. Self-rated physical function scores showed greater improvement in the GET group than in the Control group — but not self-rated fatigue scores – which suggests GET had a modest effect on patients’ perceptions of their physical function, but did not do much to alter symptom perceptions. Conversely, self-rated fatigue showed greater improvement in the CBT group than in Controls – but not physical function – which suggests CBT elicits modest reductions in symptom-focusing, but does not do much to improve patients’ confidence in their physical capacities.

Second, when recovery rates were calculated using the definition specified in the published protocol, these were extremely low across the board, and not significantly greater in the CBT or GET groups than in the Control group. Neither an intent-to-treat nor an available case analysis yielded a significant benefit for these therapies over conventional medical care. Again, we were unable to incorporate a number of stratification variables into this analysis, but it is unlikely that the result would be different had we done so.

With respect to long-term outcomes, the investigators’ original analysis did not reveal any significant effects of treatment allocation on self-reported fatigue and physical function at long-term follow-up [7]. They suggested this null effect may have been due to the confounding effects of post-trial therapy. Our informal re-examination of the long-term follow-up results provide no support for this suggestion. We found that even when patients who received post-trial CBT or GET are excluded, there is still no evidence of any long-term treatment-related benefits – not even a trend in the hypothesised direction. Of course, our analyses were informal. Ideally, we would have replicated the analysis reported in [7] for this patient subset, which included all the covariates listed in that analysis ([7], p. 1068), such as fatigue and physical function scores at 52 weeks, time of follow-up, trial centre and disease caseness. This was not possible on the data available. However, until better evidence becomes available, there is no reason to believe that post-trial therapy can offer a viable explanation for the absence of treatment effects at long-term follow-up.

One major problem for the PACE trial is that it was originally designed around a highly optimistic view of the therapeutic benefits of CBT and GET. Drawing on results from previous, smaller trials, the PACE investigators estimated that CBT would be likely to yield an improvement rate some six times greater than medical care alone, and GET would yield a rate five times greater [20]. These expectations formed the basis of the power calculations for the trial. But unfortunately, the improvement rates for CBT and GET participants - when compared with Control participants - fell markedly short of those expectations. So it is perhaps not surprising that an analysis of the binary improvement data alone was insufficient to detect any statistically reliable effects. In this context, it would have been perfectly acceptable first to report the protocol-specified primary outcome analysis, and then to explore the data using methods that are more sensitive to smaller effects – for example, analysis of the individual, continuous outcome measures. However, instead, the researchers chose to omit the former analysis altogether, and report only the latter. They then reported improvement rates based on an entirely new, and much more generous, definition of improvement. In sum, the analyses that were the least complimentary to CBT and GET never appeared in the published reports; the analyses that showed these interventions in a more favourable light were the only ones to be published.

As we have already pointed out, the timing of the change to the primary outcome – several months after trial completion - was highly problematic. There was also insufficient independent justification for making the change. For reasons that are never made clear, investigators had suddenly taken the view that “…a composite measure would be hard to interpret, and would not allow us to answer properly our primary questions of efficacy (i.e. comparing treatment effectiveness at reducing fatigue and disability).” ([13], p. 25). Certainly, the separate analysis of the two continuous measures provides useful additional information, but this does not justify abandoning the originally planned outcome. Further, the protocol already included measures of specific improvement rates in self-rated fatigue and physical function, and it is not clear why these were abandoned in favour of the new measure.

Turning now to the recovery rates, the late changes to the definition of recovery made it much easier for a patient to qualify as recovered. These changes were quite substantial. For example, the minimum physical function score required to qualify as recovered was reduced from 85 to 60, which is close to the mean score for patients with Class II congestive heart failure (57/100 [21]), and lower than the score required for trial entry (65/100). Also, on the fatigue criterion, a patient could now count as “recovered” despite reporting continuing fatigue on as many as seven out of the 11 fatigue questionnaire items*,* a level that substantially overlaps with that required for trial entry. Again, these changes operated to favour the study hypotheses. They enabled the researchers to make the claim that CBT and GET were significantly more likely to lead to recovery than conventional medical care (the original recovery definition would have yielded a null result), and to declare that at least “a fifth” of participants recover with CBT and GET [4, 22]. Neither claim could have been made if the original definition of recovery had been used.

Again, the timing of the change to the recovery definition – over a year after the trial was completed - is highly problematic. Also, an adequate justification for the change is yet to be provided. In their 2013 publication on recovery rates, the researchers argued that the normal ranges for some key scores were wider than previously thought, which would justify classing more participants as “recovered” on these measures [4]. However, we have recently shown that when the chronically ill and the very old were excluded from the relevant reference samples, and where correct statistics were applied to determine appropriate cut-off values, the normal ranges are, if anything, narrower than previously believed [17]. Consequently, this argument does not stand up to scrutiny (see [17] for further details).

Several other arguments have been presented in defence of these changes [23, 24]. One was that since there is no agreed definition of recovery, the new modified one is just as good as the original (the original definition “simply makes different assumptions” [24], p. 289). This argument fails to explain why the definition was changed in the first place. If both definitions are indeed equally good, then the one to be preferred is surely the one that was specified in advance, before any of the results were known. Another argument was that the recovery rates obtained with the modified definition were numerically similar to those found in some previous trials of CBT for CFS [23]. However, these other trials used entirely different definitions of recovery, so are not relevant here. One final argument was that the original definition of recovery was simply “too stringent to capture clinically meaningful recovery” [23]. However, the only supporting evidence for this statement comes from the disappointing recovery rates in the PACE trial itself; no *independent* justification is offered. Clearly, a strong concept like recovery must be operationalised carefully. Physicians and lay people understand this term to mean a return to good health [25], and any definition must preserve this core meaning. If anything, the original protocol-specified definition was rather generous, and may have identified some individuals that had not recovered in the plain English sense of the word. For example, on the primary physical function measure (the SF36), it was possible to score in the bottom decile for working age individuals with no long-term illness or disability, and still count as recovered on that criterion [17]. The definition also did not require evidence of an ability to return to work or other premorbid activities, even though these are very important components of what recovery means to patients. There was certainly no justification for further loosening that definition. In sum, none of the trial investigators’ arguments adequately justified the late changes to the recovery definition. More detailed discussion of these issues can be found elsewhere [26].

Turning now to long-term follow-up, the original publication of the long-term follow-up data reported no significant differences amongst treatment groups at this time point [7]. However, the authors dismissed their own finding, arguing that many participants received additional post-trial therapy which might have operated to obscure group differences. Instead, they based their main conclusion on comparisons *between time points*. For example, the first line of the Discussion reads: “The main finding of this long-term follow-up study of the PACE trial participants is that the beneficial effects of the rehabilitative CBT and GET therapies on fatigue and physical functioning observed at the final 1 year outcome of the trial *were maintained* at long-term follow-up 2·5 years from randomisation.” ([7] p. 1072, Italics added). This conclusion is repeated in the Abstract. The decision to lead with this conclusion again operated to show the findings in a more positive light than would have been possible based on their own primary between-groups analysis. The informal analyses we presented here provide no support for the investigators’ claim that post-trial therapy contaminated the long-term outcome data. Of course, our analyses did not include important potentially confounding variables that might differ amongst trial arms, and such a comprehensive analysis might possibly produce a different result. However, until there is positive evidence to suggest that this is the case, the conclusion we must draw is that PACE’s treatment effects are not sustained over the long term, not even on self-report measures. CBT and GET have no long-term benefits at all. Patients do just as well with some good basic medical care.

### Overall evaluation of the trial

Some notable strengths of the PACE study included the large sample size (determined a priori using power analysis [1]), the random allocation of patients to treatment arms, the use of a well-formulated protocol to minimise drop-outs, and the reporting of the full CONSORT trial profile (including detailed information about missing data). The incorporation of an active comparison group - Adaptive Pacing Therapy - also provided a useful secondary control for factors such as overall therapy time and patient-therapist alliance. It is worth pointing out that results for this group were not significantly different from those for the Control group on any of the measures considered in this paper. Other strengths were that each therapy group received a substantial dose of therapy, and standardised manuals ensured comparability of treatments across centres and therapists. Finally, a wide range of outcomes was measured, including several objective measures, as well as various adverse events measures.

However, despite these strengths, the design, analysis and reporting of the results introduced some significant biases. We have already discussed some of the biases that were introduced at the analysis and reporting stage. Several key results that showed CBT and GET in less than favourable light were omitted and replaced with new ones that appeared more favourable to the treatments. These changes were made at a late stage in the trial, and we have argued here that none had sufficient independent justification. In reality, the effects of CBT and GET were very modest - and not statistically reliable overall if we apply procedures very close to those specified in the original published protocol.

Another source of bias arose from the trial’s heavy reliance on self-reports from participants who were aware of their treatment allocation. Clearly, in a behavioural intervention trial, full blinding is not possible. Nevertheless, it is the researchers’ responsibility to consider the possible effects of lack of blinding on outcomes, and to ensure such factors are insufficient to account for any apparent benefits. A trial that is not blinded, self-reported outcomes in particular can produce highly inflated estimates of treatment-related benefits [27, 28]. A recent meta-analysis of clinical trials for a range of disorders found that when patients were not blinded to their treatment allocation, their self-reported improvement on the treatment of interest was inflated by 0.56 standard deviations, on average, when compared to a corresponding blinded phase of the same trial [29]. In contrast, observer-rated measures of improvement were not significantly affected by blinding. Given this discrepancy in the effects of blinding on subjective and objective measures, it appears unlikely that these effects reflect genuine health benefits. Amore plausible explanation is that they are expectation-related artefacts – for example, they reflect the operation of attentional biases that favour the reporting of events consistent with one’s expectations [30], or recall/confirmation biases that enhance recollection for expectation-consistent events [31].

The PACE investigators have argued that expectancy effects alone cannot account for the positive self-reported improvements, because at the start of treatment, patients’ expectations of improvement were not greater in the CBT and GET groups than in the other groups [2, 23]. However, they fail to point out that CBT and GET participants were *primed during treatment* to expect improvement. The manual given to CBT participants at the start of treatment proclaimed CBT to be “a powerful and safe treatment which has been shown to be effective in... CFS/ME” ([32], p. 123). The GET participants’ manual described GET as “one of the most effective therapy strategies currently known” ([33], p. 28). Both interventions emphasised that faithful adherence to the programme could lead to a full recovery. Such messages — from an authoritative source — are likely to have substantially raised patients’ expectations of improvement. Importantly, no such statements were given to the other treatment groups. When we add to this the fact that the CBT programme, and to a lesser extent GET, was designed to reduce “symptom focusing”, which may have further influenced self-report behaviour in the absence of genuine improvement [27, 34], these findings start to look very worrying indeed.

A further cause for concern in the PACE trial was that the two primary self-report measures appear to behave in different ways depending upon the intervention. Our analysis based of the protocol-specified outcomes indicated that GET produces modest enhancements in patients’ perceived physical function, but has little effect on symptom perception. Conversely, CBT improved symptom perception – specifically, self-rated fatigue scores – but had little effect on perceived physical function. If these interventions were operating to create a genuine underlying change in illness status, we would expect change on one measure to be accompanied by change on the other.

Given the high risk of participant response bias in this study, it was therefore crucial to demonstrate accompanying improvement on more objective measures. However, only one such measure showed a treatment effect. On the six-minute walking test, the originally-reported available case analysis found that GET participants walked reliably farther than Control participants at the primary, 52-week endpoint. However, after *an entire year,* this group walked an average of just 67 m farther than baseline, and around 30 m farther than Controls. To put this in context, a sample of Class II chronic heart failure patients with similar baseline walking distances increased their distance by an average of 141 m after *only three weeks* of a gentle graded exercise programme [35].

No other objective measures yielded significant treatment effects. Most notably, treatment did not affect aerobic fitness, measured using a step test. If GET had genuinely improved participants’ physical function and levels of activity, these improvements should have been clearly evident on fitness measures taken a full year after trial commencement. Treatment also did not affect time lost from work [3]. There was ample opportunity for improvement here: during the six months preceding the trial, 83% of participants were either in work or would have worked if able (based on the number reporting lost work days). This suggests they could have immediately increased their hours if their health had permitted. Finally, the percentage of participants receiving government benefits or income protection actually *increased* over the treatment period for all groups [3]. It is concerning that these negative findings were not even published until years after the primary results had been reported, so these inconsistencies are not immediately apparent to the reader. For example, the crucial fitness results were not published until four years after the primary outcomes. The investigators dismissed most of these measures as unimportant or unreliable; they did not consider them valuable as a means of estimating the degree of bias inherent in their self-report outcomes.

The absence of evidence for treatment-related recovery is an additional, serious concern for the trial. CBT and GET were not seen as adjunct treatments that might relieve a little distress. Rather, they were seen as capable of *reversing the very behaviours and cognitions responsible for CFS*. The behavioural-deconditioning model, on which the treatments were based, assumes that there is no underlying disease process in CFS, and that patients’ concerns about exercise are merely “fearful cognitions” that need addressing ([36], p. 47–8). Participants in some trial arms were even told that “there is nothing to stop your body from gaining strength and fitness” ([32], p. 31). If this model of CFS were correct, and if the treatments were operating as hypothesised, then some participants that duly followed the programme should have returned to the levels of health and physical function, that they enjoyed prior to illness onset. Therefore, the rates of recovery in the CBT and GET groups should have been significantly and reliably higher than in the Control group, irrespective of the method used to define recovery. This was not the case.

The failure of CBT and GET to “reverse” CFS is perhaps not so surprising when we consider recent exercise physiology studies. CFS patients have shown various physical abnormalities when tested 24 h after exertion (reduced VO_2_max and/or anaerobic thresholds; for a review, see [37]). These abnormalities are not seen in sedentary, healthy adults or even in patients with cardiovascular disease, and therefore cannot be attributed to deconditioning alone. Such findings call into question the core assumption of the behavioural/deconditioning model that there is no ongoing disease process. If there is a rational basis for patients’ concerns over exercise, encouraging them to push through symptoms may be harmful, and recasting patients’ concerns as dysfunctional may cause additional, psychological harm.

Turning now to safety issues, there were few group differences in the incidence of adverse events, and the researchers concluded that both CBT and GET were safe for people with CFS. This finding – particularly that relating to GET - contrasts markedly with findings from informal surveys conducted by patient organisations [38, 39]. In these surveys, between 33% and 79% of respondents report worsened health as a result of having participated in some form of graded exercise programme (weighted average across 11 different surveys: 54% [39]). Of course, in such surveys, participant self-selection may operate to enhance the reporting rates for adverse outcomes. However, this finding is so consistent, and the number of participants surveyed is so large (upwards of 10,000 cases), that it cannot be entirely dismissed. One likely reason for the discrepancy between PACE’s findings and those of patient surveys is the conservative approach used in PACE’s GET programme. Patients were encouraged to increase activity *only if it provoked no more than mild symptoms* [40]. Unfortunately, compliance with the activity recommendations was not directly assessed: actigraphy data were collected only at trial commencement [1] and never reported. This is a significant omission, since there is evidence that graded exercise therapies are not always successful in actually increasing CFS patients’ activity levels [41]. Even those who comply with exercise goals may reduce other activities to compensate [42]. The lack of improvement in fitness levels in PACE’s GET group does suggest that participants may not have substantially increased their activity levels, even over the course of an entire year. Also, even though the majority of GET participants chose walking as their primary activity [2], this group demonstrated an average increase in walking speed of only 10% after an entire year (increases of 50% or more have been observed in other patient populations [35]). Given these features, it is inappropriate to generalise the safety findings from PACE to graded activity programmes more widely, especially as they are currently implemented in clinical settings.

## Conclusion

In conclusion, the various treatment effects reported in the PACE trial were modest, almost entirely confined to self-report measures, and did not endure beyond two years. If one were to ask, “Given the procedures used here, what pattern of results would we expect if these therapies *did not produce genuine change*?” the answer would be, “Modest, short-lived changes in self-report behaviour unaccompanied by objectively measurable changes” — a pattern much like the one obtained. Given the size and power of the PACE trial, it seems unlikely that further research based on these treatments will yield more favourable results. Indeed, another large parallel trial that involved home-based therapy, described as PACE’s “sister trial”([43]), also yielded null outcomes at its primary endpoint [44, 45]. The time has come to look elsewhere for effective treatments. Current major NIH research initiatives include a large intramural study of post-infectious CFS, which aims to examine the pathophysiology of this phenotype specifically [46], and a systematic investigation of inflammatory markers (both peripheral and CNS) in CFS, and how they are influenced by exertion [47]. Such initiatives have the potential to play a key role in generating new treatment paradigms.

## Abbreviations

CBT: Cognitive behavioural therapy
CDC: US Centers for Disease Control
CFQ: Chalder Fatigue Questionnaire
CFS: Chronic fatigue syndrome
CI(s): Confidence interval(s)
CNS: Central nervous system
CONSORT: Consolidated Standards of Reporting Trials
FOI(A): United Kingdom Freedom of Information (Act)
GET: Graded exercise therapy
ME: Myalgic encephalomyelitis
NIH: US National Institutes of Health
SF-36: Short-form
VO2max: Maximal oxygen uptake

## References

[1] White PD, Sharpe MC, Chalder T, DeCesare JC, Walwyn R. Protocol for the PACE trial: a except to say it was “before the analysis occurredcognitive behaviour therapy, and graded exercise as supplements to standardised specialist medical care versus standardised specialist medical care alone for patients with the chronic fatigue syndrome/myalgic encephalomyelitis or encephalopathy. BMC Neurol 2007;7:1. 10.1186/1471-2377-7-6 PMC2147058 17397525

[2] White PD, Goldsmith KA, Johnson AL, Potts L (2011). Comparison of adaptive pacing therapy, cognitive behaviour therapy, graded exercise therapy, and specialist medical care for chronic fatigue syndrome (PACE): a randomised trial. Lancet.

[3] McCrone P, Sharpe M, Chalder T, Knapp M, Johnson AL, Goldsmith KA, White PD (2012). Adaptive pacing, cognitive behaviour therapy, graded exercise, and specialist medical care for chronic fatigue syndrome: a cost-effectiveness analysis. PLoS One.

[4] White PD, Goldsmith K, Johnson AL, Chalder T, Sharpe M (2013). Recovery from chronic fatigue syndrome after treatments given in the PACE trial. Psychol Med.

[5] Dougall D, Johnson A, Goldsmith K, Sharpe M, Angus B, Chalder T, White P (2014). Adverse events and deterioration reported by participants in the PACE trial of therapies for chronic fatigue syndrome. J Psychosom Res.

[6] Chalder T, Goldsmith KA, White PD, Sharpe M, Pickles AR (2015). Rehabilitative therapies for chronic fatigue syndrome: a secondary mediation analysis of the PACE trial. Lancet Psychiatry..

[7] Sharpe M, Goldsmith KA, Johnson AL, Chalder T, Walker J, White PD (2015). Rehabilitative treatments for chronic fatigue syndrome: long-term follow-up from the PACE trial. Lancet Psychiatry.

[8] United Kingdom National Institute for Health and Care Excellence, Centre for Clinical Practice (2011). Review of clinical guideline (CG53) – chronic fatigue syndrome/ myalgic encephalomyelitis.

[9] Queen Mary University of London (QMUL): Statement: disclosure of PACE trial data under the freedom of information act. 2016. http://www.qmul.ac.uk/media/news/items/smd/181216.html Accessed 1 Oct 2016.

[10] Sharpe MC, Archard LC, Banatvala JE, Borysiewicz LK, Clare AW, David A, Edwards RH, Hawton KE, Lambert HP, Lane RJ (1991). A report—chronic fatigue syndrome: guidelines for research. J R Soc Med.

[11] Ware JE, Sherbourne CD (1992). The MOS 36-item short-form health survey (SF-36): I. Conceptual framework and item selection. Med Care.

[12] Chalder T, Berelowitz G, Pawlikowska T, Watts L, Wessely S, Wright D, Wallace EP (1993). Development of a fatigue scale. J Psychosom Res.

[13] White PD, Chalder T, Sharpe M (2015). The planning, implementation and publication of a complex intervention trial for chronic fatigue syndrome: the PACE trial. BJPsych Bull.

[14] Walwyn R, Potts L, McCrone P, Johnson AL, DeCesare JC, Baber H, Goldsmith K, Sharpe M, Chalder T, White PD (2013). A randomised trial of adaptive pacing therapy, cognitive behaviour therapy, graded exercise, and specialist medical care for chronic fatigue syndrome (PACE): statistical analysis plan. Trials.

[15] Queen Mary University of London (QMUL): Pace trial - published papers. 2016. http://www.wolfson.qmul.ac.uk/current-projects/pace-trial#published-papers Accessed 6 Dec 2017.

[16] Queen Mary University of London (QMUL): Pace Trial. 2016. http://www.wolfson.qmul.ac.uk/current-projects/pace-trial#patients Accessed 23 Sept 2017.

[17] Wilshire C, Kindlon T, Matthees A, McGrath S (2017). Can patients with chronic fatigue syndrome really recover after graded exercise or cognitive behavioural therapy? A critical commentary and preliminary re-analysis of the PACE trial. Fatigue.

[18] Matthees A, Kindlon T, Maryhew C, Stark P, Levin B. A preliminary analysis of ‘recovery’ from chronic fatigue syndrome in the PACE trial using individual participant data. Virology Blog. 2016; http://www.virology.ws/wp-content/uploads/2016/09/preliminary-analysis.pdf. Accessed 1 Oct 2016

[19] Goldsmith KA, White PD, Chalder T, Johnson AL, Sharpe M. The PACE trial: analysis of primary outcomes using composite measures of improvement: Unpublished report, Queen Mary University of London; 2016. http://www.wolfson.qmul.ac.uk/images/pdfs/pace/PACE_published_protocol_based_analysis_final_8th_Sept_2016.pdf. Accessed 1 Oct 2016

[20] White et al. PACE trial protocol: Final version 5.0, 01.02.2006. http://www.meactionuk.org.uk/FULL-Protocol-SEARCHABLE-version.pdf. Accessed 1 Oct 2016.

[21] Juenger J, Schellberg D, Kraemer S, Haunstetter A, Zugck C, Herzog W, Haass M (2002). Health related quality of life in patients with congestive heart failure: comparison with other chronic diseases and relation to functional variables. Heart.

[22] King’s College London. CBT for chronic fatigue syndrome. https://www.kcl.ac.uk/ioppn/about/difference/22-CBT-for-chronic-fatigue-syndrome.aspx. Accessed 23 Sept 2017.

[23] Sharpe M, Chalder T, Johnson AL, Goldsmith KA, White PD (2017). Fatigue.

[24] Chalder T, White PD, Sharpe M, Mitchell AJ (2017). Controversy over exercise therapy for chronic fatigue syndrome: continuing the debate. BJPsych Advances.

[25] Devendorf AR, Jackson CT, Sunnquist M, A Jason L (2017). Defining and measuring recovery from myalgic encephalomyelitis and chronic fatigue syndrome: the physician perspective. Disabil Rehabil.

[26] Wilshire C, Kindlon T, McGrath S (2017). PACE trial claims of recovery are not justified by the data: a rejoinder to Sharpe, Chalder, Johnson, goldsmith and white (2017). Fatigue.

[27] Lilienfeld SO, Ritschel LA, Lynn SJ, Cautin RL, Latzman RD (2014). Why ineffective psychotherapies appear to work: a taxonomy of causes of spurious therapeutic effectiveness. Perspect Psychol Sci.

[28] Hróbjartsson A, Kaptchuk TJ, Miller FG (2011). Placebo effect studies are susceptible to response bias and to other types of biases. J Clin Epidemiol.

[29] Hróbjartsson A, Emanuelsson F, Thomsen AS, Hilden J, Brorson S (2014). Bias due to lack of patient blinding in clinical trials. A systematic review of trials randomizing patients to blind and nonblind sub-studies. Int J Epidemiol.

[30] Allan LG, Siegel S (2002). A signal detection theory analysis of the placebo effect. Eval Health Prof.

[31] Rothbart M, Evans M, Fulero S (1979). Recall for confirming events: memory processes and the maintenance of social stereotypes. J Exp Soc Psychol.

[32] Burgess M, Chalder T (2004). PACE manual for participants: cognitive behavioural therapy.

[33] Bavinton J, Dyer N, White PD (2004). PACE manual for participants: graded exercise therapy.

[34] Howard GS (1980). Response-shift bias: a problem in evaluating interventions with pre/post self-reports. Eval Rev.

[35] Meyer K, Schwaibolda M, Westbrook S, Beneke R, Hajric R, Lehmann M, Roskamm H (1997). Effects of exercise training and activity restriction on 6-minute walking test performance in patients with chronic heart failure. Am Heart J.

[36] Burgess M, Chalder T. PACE manual for therapists: cognitive behavioural therapy. 2004. http://www.wolfson.qmul.ac.uk/images/pdfs/5.get-therapist-manual.pdf. Accessed 1 Oct 2016.

[37] Keller BA, Pryor JL, Giloteaux L (2014). Inability of myalgic encephalomyelitis/chronic fatigue syndrome patients to reproduce VO2peak indicates functional impairment. J Transl Med.

[38] Kindlon T (2011). Reporting of harms associated with graded exercise therapy and cognitive behavioural therapy in myalgic encephalomyelitis/chronic fatigue syndrome. Bull IACFS ME.

[39] Geraghty K, Hann M, Kurtev S (2017). Myalgic encephalomyelitis/chronic fatigue syndrome patients’ reports of symptom changes following cognitive behavioural therapy, graded exercise therapy and pacing treatments: analysis of a primary survey compared with secondary surveys. J Health Psychol.

[40] Bavinton J, Darbyshire L, White PD (2004). PACE manual for therapists: graded exercise therapy for CFS/ME: manual for therapists.

[41] Wiborg JF, Knoop H, Stulemeijer M, Prins JB, Bleijenberg G (2010). How does cognitive behaviour therapy reduce fatigue in patients with chronic fatigue syndrome? The role of physical activity. Psychol Med.

[42] Friedberg F (2002). Does graded activity increase activity? A case study of chronic fatigue syndrome. J Behav Ther Exp Psychiatry.

[43] PACE Participants’ Newsletter Issue 1. 2006. http://www.wolfson.qmul.ac.uk/images/pdfs/participantsnewsletter1.pdf. Accessed 1 Oct 2016.

[44] Wearden AJ, Riste L, Dowrick C, Chew-Graham C, Bentall RP, Morriss RK, Peters S, Dunn G, Richardson G, Lovell K, Powell P (2006). Fatigue intervention by nurses evaluation–the FINE trial. A randomised controlled trial of nurse led self-help treatment for patients in primary care with chronic fatigue syndrome: study protocol. [ISRCTN74156610]. BMC Med.

[45] Wearden AJ, Dowrick C, Chew-Graham C, Bentall RP, Morriss RK, Peters S, Riste L, Richardson G, Lovell K, Dunn G (2010). Nurse led, home based self help treatment for patients in primary care with chronic fatigue syndrome: randomised controlled trial. BMJ.

[46] National Institutes of Health. NIH intramural study on Myalgic encephalomyelitis/chronic fatigue syndrome. 2016. http://mecfs.ctss.nih.gov. Accessed 1 Oct 2016.

[47] National Institutes of Health. Project information: project 1U54NS105541–01. 2016. https://projectreporter.nih.gov/project_info_description.cfm?aid=9479859&icde=36166731&ddparam=&ddvalue=&ddsub=&cr=3&csb=default&cs=ASC&pball=. Accessed 20 Dec 2017.

[48] Loge JH, Ekeberg Ø, Kaasa S (1998). Fatigue in the general Norwegian population: normative data and associations. J Psychosom Res.

[49] Bowling A, Bond M, Jenkinson C, Lamping DL (1999). Short form 36 (SF-36) health survey questionnaire: which normative data should be used? Comparisons between the norms provided by the omnibus survey in Britain, the health survey for England and the Oxford healthy life survey. J Public Health.

[50] Guy W. Clinical global impression scale. The ECDEU assessment manual for psychopharmacology-revised Volume DHEW Publ No ADM. 1976;76(338):218–212.

[51] Reeves WC, Lloyd A, Vernon SD (2003). Identification of ambiguities in the 1994 chronic fatigue syndrome research case definition and recommendations for resolution. BMC Health Serv Res.

[52] Tyrrell DAJ (1994). Report from the National Task Force on chronic fatigue syndrome (CFS), post viral fatigue syndrome (PVFS) and myalgic encephalomyelitis (ME).

